# 2,2-Difluoro-4-phenyl-1,3,2-dioxaborolo[4,5-*c*]chromen-5-ium-2-ide

**DOI:** 10.1107/S1600536810048919

**Published:** 2010-11-30

**Authors:** Michał Wera, Alexander D. Roshal, Jerzy Błażejowski

**Affiliations:** a Faculty of Chemistry, University of Gdańsk, J. Sobieskiego 18, 80-952 Gdańsk, Poland; b Institute of Chemistry, V.N. Karazin National University, Svobody 4, 61077 Kharkiv, Ukraine

## Abstract

In the crystal, the inversely oriented mol­ecules of the title compound, C_15_H_9_BF_2_O_3_, form stacks along the *a* axis *via* π–π inter­actions between parallel phenyl­chromenium fragments. Linked by a network of C—H⋯F inter­actions, the stacks form layers in the *ac* plane that are dispersively stabilized in the crystal structure. Two F atoms bonded to the B atom are located in the plane perpendicular to the planar skeleton of the mol­ecule made rigid by two intra­molecular C—H⋯O inter­actions.

## Related literature

For general background to 3-hy­droxy-2-phenyl-4*H*-chromene-4-one (flavonol) and its derivatives, see: Kukharenko & Avramenko (2001 ▶); Petković *et al.* (2010 ▶); Roshal *et al.* (1998 ▶, 2003 ▶); Sytnik *et al.* (1994 ▶). For related structures, see: Belogh-Hergovich *et al.* (1999 ▶); Farina *et al.* (1995 ▶); Kaizer *et al.* (2007 ▶); Okabe *et al.* (2003 ▶). For inter­molecular inter­actions, see: Choudhury & Guru Row (2004 ▶); Hunter *et al.* (2001 ▶); Novoa *et al.* (2006 ▶); Thalladi *et al.* (1998 ▶). For the synthesis, see: Roshal *et al.* (2002 ▶).
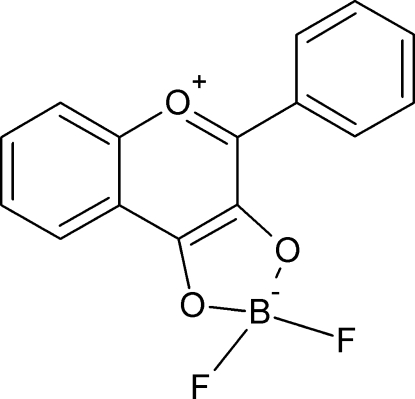


## Experimental

### 

#### Crystal data

C_15_H_9_BF_2_O_3_
*M* *_r_* = 286.03
Triclinic, 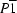

*a* = 7.1969 (10) Å
*b* = 9.7054 (11) Å
*c* = 9.9986 (15) Å
α = 74.310 (11)°
β = 75.931 (13)°
γ = 71.296 (11)°
*V* = 627.43 (15) Å^3^
*Z* = 2
Mo *K*α radiation
μ = 0.12 mm^−1^
*T* = 295 K
0.6 × 0.02 × 0.02 mm

#### Data collection

Oxford Diffraction Gemini R Ultra Ruby CCD diffractometer
Absorption correction: multi-scan (*CrysAlis RED*; Oxford Diffraction, 2008 ▶) *T*_min_ = 0.945, *T*_max_ = 0.979
4848 measured reflections
2218 independent reflections
996 reflections with *I* > 2σ(*I*)
*R*_int_ = 0.034

#### Refinement

*R*[*F* ^2^ > 2σ(*F* ^2^)] = 0.058
*wR*(*F* ^2^) = 0.234
*S* = 1.10
2218 reflections
190 parameters
H-atom parameters constrained
Δρ_max_ = 0.22 e Å^−3^
Δρ_min_ = −0.24 e Å^−3^

### 

Data collection: *CrysAlis CCD* (Oxford Diffraction, 2008 ▶); cell refinement: *CrysAlis RED* (Oxford Diffraction, 2008 ▶); data reduction: *CrysAlis RED*; program(s) used to solve structure: *SHELXS97* (Sheldrick, 2008 ▶); program(s) used to refine structure: *SHELXL97* (Sheldrick, 2008 ▶); molecular graphics: *ORTEP-3* (Farrugia, 1997 ▶); software used to prepare material for publication: *SHELXL97* and *PLATON* (Spek, 2009 ▶).

## Supplementary Material

Crystal structure: contains datablocks global, I. DOI: 10.1107/S1600536810048919/ng5071sup1.cif

Structure factors: contains datablocks I. DOI: 10.1107/S1600536810048919/ng5071Isup2.hkl

Additional supplementary materials:  crystallographic information; 3D view; checkCIF report

## Figures and Tables

**Table 1:** Hydrogen-bond geometry (Å, °)

*D*—H⋯*A*	*D*—H	H⋯*A*	*D*⋯*A*	*D*—H⋯*A*
C2—H2⋯O3	0.93	2.37	2.700 (6)	100
C6—H6⋯O1	0.93	2.29	2.967 (6)	129
C11—H11⋯F1^i^	0.93	2.44	3.373 (6)	177

Symmetry code: (i) 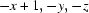.

**Table 2:** π–π inter­actions (Å,°) *Cg*1, *Cg*2 and *Cg*3 are the centroids of the O3/C7–C10/C15, C10–C15 and C1–C6 rings, respectively. *CgI*⋯*CgJ* is the distance between ring centroids. The dihedral angle is that between the planes of the rings *I* and *J. CgI*_Perp is the perpendicuar distance of *CgI* from ring *J. CgI*_Offset is the distance between *CgI* and the perpendicular projection of *CgJ* on ring *I*.

*I*	*J*	*CgI*⋯*CgJ*	Dihedral angle	*CgI*_Perp	*CgI*_Offset
1	1^ii^	3.512 (3)		3.344 (2)	1.076 (2)
1	3^iii^	3.572 (3)	2.3 (3)	3.450 (2)	0.956 (2)
2	3^ii^	3.970 (3)	4.3 (3)	3.342 (2)	2.143 (2)
2	3^iii^	3.925 (3)	4.3 (3)	3.472 (2)	1.831 (2)
3	1^iii^	3.571 (3)	2.3 (3)	3.428 (2)	1.000 (2)
3	2^ii^	3.970 (3)	4.3 (3)	3.492 (2)	1.889 (2)
3	2^iii^	3.925 (3)	4.3 (3)	3.404 (2)	1.954 (2)

Symmetry codes: (ii) 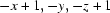; (iii) 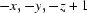.

**Fig. 1.**
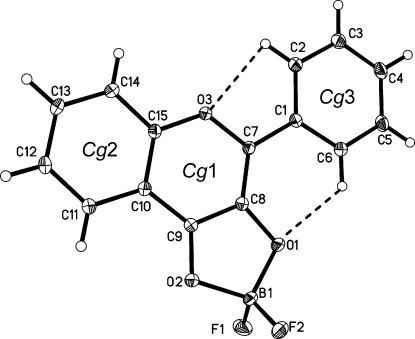
The molecular structure of the title compound showing the atom-labeling scheme. Displacement ellipsoids are drawn at the 25% probability level, and H atoms are shown as small spheres of arbitrary radius. Cg1, Cg2 and Cg3 denote the ring centroids. The C–H···O hydrogen bonds are represented by dashed lines.

**Fig. 2.**
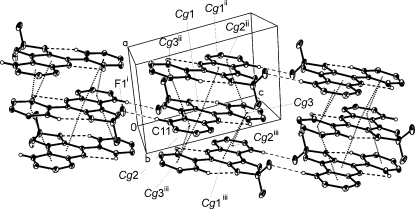
The arrangement of the molecules in the crystal structure. The C–H···O and C–H···F interactions are represented by dashed lines, the π–π contacts by dotted lines. H atoms not involved in interactions have been omitted. [Symmetry codes: (i) –x + 1, –y, –z; (ii) –x + 1, –y, –z + 1; (iii) –x, –y, –z + 1.]

**Fig. 3.**
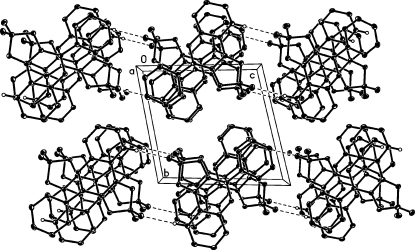
Molecular stacks in the crystal structure, viewed along the a axis. The C–H···F interactions are represented by dashed lines. H atoms not involved in interactions have been omitted.

## supplementary crystallographic information

### Comment

3-Hydroxy-2-phenyl-4*H*-chromene-4-one (flavonol) and its derivatives have been investigated for a long time owing to their unique spectral properties emerging from the excited state intramolecular proton transfer occurring in them (Sytnik *et al.*, 1994). These compounds turned out to be convenient analytical probes because of their considerable ability to complex various chemical entities (molecules, ions) (Roshal *et al.*, 1998; Roshal *et al.*, 2003; Petković *et al.*, 2010). The latter property was the reason for turning our attention to the possibility of applying of flavonol as an analytical spectral probe for boron compounds (Roshal *et al.*, 2002). As part of these investigations we wanted to see how flavonol behaved in the presence of BF_3_. Thus we mixed both reagents in dichloromethane, expecting to obtain a molecular complex of flavonol and BF_3_. The structure of the crystalline product that was actually separated is presented here. It appears that not complexation but condensation of the two reagents, accompanied by the release of HF, takes place (Kukharenko & Avramenko, 2001) and a molecule of a formally zwitterionic canonical structure was produced. Crystal structures of various flavonol complexes have so far been reported (Farina & Yamin, 1995; Belogh-Hergovich *et al.*, 1999; Okabe *et al.*, 2003; Kaizer *et al.*, 2007), but none of them have contained boron.

The canonical structure of the title compound suggests that the phenylchromenium core of the molecule is aromatic. This is confirmed by analysis of the bond lengths and angles, as well as comparison of the structure determinated here with the structures of selected compounds containing flavonol units (Farina *et al.*, 1995; Belogh-Hergovich *et al.*, 1999; Okabe *et al.*, 2003; Kaizer *et al.*, 2007). Furthermore, the average deviation from planarity of the phenylchromenium core is 0.0215 (2) and that of the molecule's skeleton is 0.0373 (2). This implies that both the above-mentioned molecular fragments are planar and that two F atoms at the B atom are located in a plane perpendicular to the molecular one (the dihedral angle between the plane of the molecule's skeleton and the plane of B1–F1–F2 is 89.5 (1)°). Furthermore, two intramolecular C–H···O interactions (Table 1, Fig. 1) stiffen the phenylchromenium core, undoubtedly contributing to its planarity.

In the crystal structure, inversely oriented molecules form stacks along the *a* axis *via*π-π interactions between parallel phenylchromenium fragments (Table 2, Figs. 2 and 3). These stacks are linked by a network of C–H···F interactions arranged in layers in the *ac* plane (Table 1, Figs. 2 and 3). The above-mentioned layers are dispersively stabilized in the crystal lattice. The C–H···O (Novoa *et al.*, 2006) and C–H···F (Thalladi *et al.*(1998); Choudhury & Guru Row (2004)) interactions are of the hydrogen bond type. Like the π-π contacts, they are of an attractive nature (Hunter *et al.*, 2001).

### Experimental

The title compound was obtained during the reaction of 3-hydroxy-2-phenyl-4*H*-chromene-4-one (flavonol) with BF_3_ (Roshal *et al.*, 2002). Thus, boron trifluoride dissolved in anhydrous diethyl ether was added dropwise, with continuous stirring, to an equimolar amount of a saturated solution of flavonol in anhydrous dichloromethane. After evaporation of the solvents, the residue was recrystallized twice from *N*,*N*-dimethylformamide yielding yellow fluorescing crystals suitable for X-Ray investigations (m.p. 485 - 487 K).

### Refinement

H atoms were positioned geometrically, with C—H = 0.93 Å, and constrained to ride on their parent atoms with *U*_iso_(H) = 1.2*U*_eq_(C).

### Crystal data

**Table:**

C_15_H_9_BF_2_O_3_	*Z* = 2
*M**_r_* = 286.03	*F*(000) = 292
Triclinic, *P*1	*D*_x_ = 1.514 Mg m^−^^3^
Hall symbol: -P 1	Mo *K*α radiation, λ = 0.71073 Å
*a* = 7.1969 (10) Å	Cell parameters from 2218 reflections
*b* = 9.7054 (11) Å	θ = 3.0–25.1°
*c* = 9.9986 (15) Å	µ = 0.12 mm^−^^1^
α = 74.310 (11)°	*T* = 295 K
β = 75.931 (13)°	Needle, yellow
γ = 71.296 (11)°	0.6 × 0.02 × 0.02 mm
*V* = 627.43 (15) Å^3^	

### Data collection

**Table:**

Oxford Diffraction Gemini R Ultra Ruby CCD diffractometer	2218 independent reflections
Radiation source: Enhance (Mo) X-ray Source	996 reflections with *I* > 2σ(*I*)
graphite	*R*_int_ = 0.034
Detector resolution: 10.4002 pixels mm^-1^	θ_max_ = 25.1°, θ_min_ = 3.0°
ω scans	*h* = −8→8
Absorption correction: multi-scan (*CrysAlis RED*; Oxford Diffraction, 2008)	*k* = −10→11
*T*_min_ = 0.945, *T*_max_ = 0.979	*l* = −9→11
4848 measured reflections	

### Refinement

**Table:**

Refinement on *F*^2^	Primary atom site location: structure-invariant direct methods
Least-squares matrix: full	Secondary atom site location: difference Fourier map
*R*[*F*^2^ > 2σ(*F*^2^)] = 0.058	Hydrogen site location: inferred from neighbouring sites
*wR*(*F*^2^) = 0.234	H-atom parameters constrained
*S* = 1.10	*w* = 1/[σ^2^(*F*_o_^2^) + (0.0787*P*)^2^ + 0.4107*P*] where *P* = (*F*_o_^2^ + 2*F*_c_^2^)/3
2218 reflections	(Δ/σ)_max_ < 0.001
190 parameters	Δρ_max_ = 0.22 e Å^−^^3^
0 restraints	Δρ_min_ = −0.24 e Å^−^^3^

### Special details

**Table:**

Geometry. All e.s.d.'s (except the e.s.d. in the dihedral angle between two l.s. planes) are estimated using the full covariance matrix. The cell e.s.d.'s are taken into account individually in the estimation of e.s.d.'s in distances, angles and torsion angles; correlations between e.s.d.'s in cell parameters are only used when they are defined by crystal symmetry. An approximate (isotropic) treatment of cell e.s.d.'s is used for estimating e.s.d.'s involving l.s. planes.
Refinement. Refinement of *F*^2^ against ALL reflections. The weighted *R*-factor *wR* and goodness of fit *S* are based on *F*^2^, conventional *R*-factors *R* are based on *F*, with *F* set to zero for negative *F*^2^. The threshold expression of *F*^2^ > σ(*F*^2^) is used only for calculating *R*-factors(gt) *etc*. and is not relevant to the choice of reflections for refinement. *R*-factors based on *F*^2^ are statistically about twice as large as those based on *F*, and *R*- factors based on ALL data will be even larger.

### Fractional atomic coordinates and isotropic or equivalent isotropic displacement parameters (Å^2^)

**Table:**

	*x*	*y*	*z*	*U*_iso_*/*U*_eq_	
B1	0.4867 (13)	−0.1835 (7)	0.2184 (8)	0.075 (2)	
O1	0.3873 (5)	−0.2005 (3)	0.3680 (3)	0.0605 (10)	
C1	0.1714 (6)	−0.1185 (5)	0.6585 (5)	0.0395 (11)	
F1	0.3869 (6)	−0.2186 (4)	0.1401 (3)	0.1025 (14)	
O2	0.4773 (5)	−0.0183 (3)	0.1743 (3)	0.0594 (10)	
C2	0.0722 (8)	−0.0564 (6)	0.7748 (5)	0.0600 (15)	
H2	0.0499	0.0447	0.7694	0.072*	
F2	0.6830 (6)	−0.2651 (4)	0.2068 (4)	0.1059 (14)	
O3	0.2079 (5)	0.1167 (3)	0.5341 (3)	0.0480 (9)	
C3	0.0071 (9)	−0.1459 (6)	0.8983 (6)	0.0680 (16)	
H3	−0.0595	−0.1045	0.9764	0.082*	
C4	0.0389 (8)	−0.2934 (6)	0.9077 (6)	0.0628 (15)	
H4	−0.0061	−0.3521	0.9919	0.075*	
C5	0.1364 (8)	−0.3559 (6)	0.7946 (6)	0.0601 (15)	
H5	0.1595	−0.4574	0.8022	0.072*	
C6	0.2010 (7)	−0.2690 (5)	0.6686 (6)	0.0535 (13)	
H6	0.2643	−0.3114	0.5907	0.064*	
C7	0.2432 (6)	−0.0265 (5)	0.5263 (5)	0.0408 (11)	
C8	0.3379 (7)	−0.0667 (5)	0.3996 (5)	0.0436 (12)	
C9	0.3902 (7)	0.0404 (5)	0.2829 (5)	0.0453 (12)	
C10	0.3501 (7)	0.1903 (5)	0.2890 (5)	0.0414 (11)	
C11	0.3933 (7)	0.3038 (5)	0.1770 (5)	0.0549 (14)	
H11	0.4525	0.2835	0.0882	0.066*	
C12	0.3484 (8)	0.4446 (6)	0.1983 (6)	0.0587 (14)	
H12	0.3739	0.5212	0.1237	0.070*	
C13	0.2639 (8)	0.4731 (5)	0.3325 (6)	0.0601 (15)	
H13	0.2371	0.5689	0.3466	0.072*	
C14	0.2191 (7)	0.3639 (5)	0.4443 (6)	0.0566 (14)	
H14	0.1636	0.3844	0.5334	0.068*	
C15	0.2586 (7)	0.2227 (5)	0.4210 (5)	0.0454 (12)	

### Atomic displacement parameters (Å^2^)

**Table:**

	*U*^11^	*U*^22^	*U*^33^	*U*^12^	*U*^13^	*U*^23^
B1	0.113 (6)	0.055 (4)	0.059 (5)	−0.037 (4)	0.023 (4)	−0.031 (4)
O1	0.090 (3)	0.0377 (19)	0.050 (2)	−0.0213 (18)	0.0070 (19)	−0.0153 (16)
C1	0.037 (3)	0.042 (3)	0.039 (3)	−0.013 (2)	−0.005 (2)	−0.007 (2)
F1	0.189 (4)	0.081 (2)	0.059 (2)	−0.073 (3)	0.000 (2)	−0.0260 (19)
O2	0.086 (3)	0.047 (2)	0.041 (2)	−0.0228 (18)	0.0087 (19)	−0.0157 (17)
C2	0.076 (4)	0.058 (3)	0.048 (3)	−0.026 (3)	0.003 (3)	−0.016 (3)
F2	0.122 (3)	0.062 (2)	0.092 (3)	−0.004 (2)	0.040 (2)	−0.025 (2)
O3	0.059 (2)	0.0383 (18)	0.043 (2)	−0.0122 (15)	0.0004 (16)	−0.0117 (16)
C3	0.093 (4)	0.076 (4)	0.037 (3)	−0.036 (3)	0.005 (3)	−0.014 (3)
C4	0.066 (4)	0.073 (4)	0.043 (3)	−0.032 (3)	−0.001 (3)	0.007 (3)
C5	0.058 (3)	0.054 (3)	0.055 (4)	−0.013 (3)	−0.004 (3)	0.003 (3)
C6	0.056 (3)	0.049 (3)	0.054 (3)	−0.016 (3)	−0.002 (3)	−0.012 (3)
C7	0.041 (3)	0.040 (3)	0.041 (3)	−0.009 (2)	−0.006 (2)	−0.010 (2)
C8	0.044 (3)	0.043 (3)	0.039 (3)	−0.009 (2)	−0.004 (2)	−0.008 (2)
C9	0.047 (3)	0.047 (3)	0.041 (3)	−0.014 (2)	0.000 (2)	−0.014 (2)
C10	0.045 (3)	0.038 (3)	0.043 (3)	−0.012 (2)	−0.005 (2)	−0.011 (2)
C11	0.065 (4)	0.050 (3)	0.049 (3)	−0.022 (3)	0.001 (3)	−0.011 (3)
C12	0.066 (4)	0.047 (3)	0.056 (4)	−0.021 (3)	−0.001 (3)	−0.001 (3)
C13	0.071 (4)	0.038 (3)	0.066 (4)	−0.010 (3)	−0.001 (3)	−0.018 (3)
C14	0.062 (3)	0.042 (3)	0.061 (4)	−0.011 (3)	0.001 (3)	−0.018 (3)
C15	0.046 (3)	0.039 (3)	0.046 (3)	−0.012 (2)	−0.002 (2)	−0.006 (2)

### Geometric parameters (Å, °)

**Table:**

B1—F1	1.349 (8)	C5—C6	1.381 (7)
B1—F2	1.375 (9)	C5—H5	0.9300
B1—O1	1.483 (7)	C6—H6	0.9300
B1—O2	1.527 (7)	C7—C8	1.377 (6)
O1—C8	1.336 (5)	C8—C9	1.402 (6)
C1—C6	1.386 (6)	C9—C10	1.405 (6)
C1—C2	1.387 (6)	C10—C11	1.396 (6)
C1—C7	1.462 (6)	C10—C15	1.395 (6)
O2—C9	1.293 (5)	C11—C12	1.363 (7)
C2—C3	1.378 (7)	C11—H11	0.9300
C2—H2	0.9300	C12—C13	1.394 (7)
O3—C7	1.351 (5)	C12—H12	0.9300
O3—C15	1.371 (5)	C13—C14	1.371 (7)
C3—C4	1.356 (7)	C13—H13	0.9300
C3—H3	0.9300	C14—C15	1.380 (6)
C4—C5	1.360 (7)	C14—H14	0.9300
C4—H4	0.9300		
			
F1—B1—F2	112.0 (5)	O3—C7—C8	117.9 (4)
F1—B1—O1	110.8 (5)	O3—C7—C1	113.2 (4)
F2—B1—O1	110.9 (6)	C8—C7—C1	128.9 (4)
F1—B1—O2	110.6 (6)	O1—C8—C7	128.2 (4)
F2—B1—O2	108.8 (5)	O1—C8—C9	111.7 (4)
O1—B1—O2	103.3 (4)	C7—C8—C9	120.1 (4)
C8—O1—B1	106.4 (4)	O2—C9—C8	111.0 (4)
C6—C1—C2	119.3 (4)	O2—C9—C10	126.5 (4)
C6—C1—C7	120.2 (4)	C8—C9—C10	122.5 (4)
C2—C1—C7	120.6 (4)	C11—C10—C15	119.6 (4)
C9—O2—B1	107.5 (4)	C11—C10—C9	125.9 (5)
C3—C2—C1	119.3 (5)	C15—C10—C9	114.6 (4)
C3—C2—H2	120.3	C12—C11—C10	119.7 (5)
C1—C2—H2	120.3	C12—C11—H11	120.2
C7—O3—C15	122.7 (4)	C10—C11—H11	120.2
C4—C3—C2	120.9 (5)	C11—C12—C13	119.7 (5)
C4—C3—H3	119.5	C11—C12—H12	120.1
C2—C3—H3	119.5	C13—C12—H12	120.1
C3—C4—C5	120.3 (5)	C14—C13—C12	121.9 (5)
C3—C4—H4	119.8	C14—C13—H13	119.1
C5—C4—H4	119.8	C12—C13—H13	119.1
C4—C5—C6	120.2 (5)	C13—C14—C15	118.2 (5)
C4—C5—H5	119.9	C13—C14—H14	120.9
C6—C5—H5	119.9	C15—C14—H14	120.9
C1—C6—C5	119.9 (5)	O3—C15—C14	116.8 (4)
C1—C6—H6	120.1	O3—C15—C10	122.2 (4)
C5—C6—H6	120.1	C14—C15—C10	120.9 (4)
			
F1—B1—O1—C8	−119.9 (5)	C1—C7—C8—C9	178.1 (4)
F2—B1—O1—C8	114.9 (4)	B1—O2—C9—C8	0.3 (6)
O2—B1—O1—C8	−1.5 (7)	B1—O2—C9—C10	−179.8 (5)
F1—B1—O2—C9	119.3 (5)	O1—C8—C9—O2	−1.4 (6)
F2—B1—O2—C9	−117.2 (5)	C7—C8—C9—O2	−180.0 (4)
O1—B1—O2—C9	0.7 (7)	O1—C8—C9—C10	178.7 (4)
C6—C1—C2—C3	0.7 (7)	C7—C8—C9—C10	0.2 (7)
C7—C1—C2—C3	−179.7 (5)	O2—C9—C10—C11	1.6 (8)
C1—C2—C3—C4	0.1 (8)	C8—C9—C10—C11	−178.5 (5)
C2—C3—C4—C5	0.1 (8)	O2—C9—C10—C15	−178.4 (5)
C3—C4—C5—C6	−1.0 (8)	C8—C9—C10—C15	1.4 (6)
C2—C1—C6—C5	−1.6 (7)	C15—C10—C11—C12	0.9 (7)
C7—C1—C6—C5	178.7 (4)	C9—C10—C11—C12	−179.1 (5)
C4—C5—C6—C1	1.8 (7)	C10—C11—C12—C13	1.5 (8)
C15—O3—C7—C8	1.5 (6)	C11—C12—C13—C14	−1.8 (8)
C15—O3—C7—C1	−178.3 (4)	C12—C13—C14—C15	−0.5 (8)
C6—C1—C7—O3	−178.3 (4)	C7—O3—C15—C14	−178.3 (4)
C2—C1—C7—O3	2.1 (6)	C7—O3—C15—C10	0.2 (7)
C6—C1—C7—C8	1.9 (7)	C13—C14—C15—O3	−178.5 (4)
C2—C1—C7—C8	−177.7 (5)	C13—C14—C15—C10	2.9 (7)
B1—O1—C8—C7	−179.8 (6)	C11—C10—C15—O3	178.3 (4)
B1—O1—C8—C9	1.8 (6)	C9—C10—C15—O3	−1.6 (7)
O3—C7—C8—O1	−179.9 (4)	C11—C10—C15—C14	−3.2 (7)
C1—C7—C8—O1	−0.2 (8)	C9—C10—C15—C14	176.8 (4)
O3—C7—C8—C9	−1.6 (6)		

### Hydrogen-bond geometry (Å, °)

**Table:**

*D*—H···*A*	*D*—H	H···*A*	*D*···*A*	*D*—H···*A*
C2—H2···O3	0.93	2.37	2.700 (6)	100
C6—H6···O1	0.93	2.29	2.967 (6)	129
C11—H11···F1^i^	0.93	2.44	3.373 (6)	177

Symmetry codes:  (i) −*x*+1, −*y*, −*z*.

### Table 2 π–π interactions (Å,°).

*Cg*1, *Cg*2 and *Cg*3 are the centroids of the O3/C7–C10/C15, C10–C15 and C1–C6 rings, respectively. Cg*I*···Cg*J* is the distance between ring centroids. The dihedral angle is that between the planes of the rings *I* and *J*. *CgI*_Perp is the perpendicuar distance of *CgI* from ring *J*. Cg*I*_Offset is the distance between *CgI* and the perpendicular projection of *CgJ* on ring *I*.

**Table:**

*I*	*J*	*CgI*···*CgJ*	Dihedral angle	*CgI*_Perp	Cg*I*_Offset
1	1^ii^	3.512 (3)	0	3.344 (2)	1.076 (2)
1	3^iii^	3.572 (3)	2.3 (3)	3.450 (2)	0.956 (2)
2	3^ii^	3.970 (3)	4.3 (3)	3.342 (2)	2.143 (2)
2	3^iii^	3.925 (3)	4.3 (3)	3.472 (2)	1.831 (2)
3	1^iii^	3.571 (3)	2.3 (3)	3.428 (2)	1.000 (2)
3	2^ii^	3.970 (3)	4.3 (3)	3.492 (2)	1.889 (2)
3	2^iii^	3.925 (3)	4.3 (3)	3.404 (2)	1.954 (2)

Symmetry codes: (ii) -*x* + 1, -*y*, -*z* + 1; (iii) -*x*, -*y*, -*z* + 1.

## References

[bb1] Belogh-Hergovich, E., Kaizer, J., Speier, G., Argay, G. & Párkányi, L. (1999). *J. Chem. Soc. Dalton Trans.* pp. 3847–3854.

[bb2] Choudhury, A. R. & Guru Row, T. N. (2004). *Cryst. Growth Des.* **4**, 47–52.

[bb3] Farina, Y., Yamin, B. M., Fun, H.-K., Yip, B.-C. & Teoh, S.-G. (1995). *Acta Cryst.* C**51**, 1537–1540.

[bb4] Farrugia, L. J. (1997). *J. Appl. Cryst.* **30**, 565.

[bb5] Hunter, C. A., Lawson, K. R., Perkins, J. & Urch, C. J. (2001). *J. Chem. Soc. Perkin Trans. 2*, pp. 651–669.

[bb6] Kaizer, J., Barath, G., Pap, J., Speier, G., Giorgi, M. & Reglier, M. (2007). *Chem. Commun.* pp. 5235–5237. 10.1039/b711864c 18060153

[bb7] Kukharenko, A. V. & Avramenko, G. V. (2001). *Russ. J. Gen. Chem.* **71**, 1562–1564.

[bb8] Novoa, J. J., Mota, F. & D’Oria, E. (2006). *Hydrogen Bonding – New Insights*, edited by S. Grabowski, pp. 193–244. The Netherlands: Springer.

[bb9] Okabe, N., Yamamoto, E. & Yasunori, M. (2003). *Acta Cryst.* E**59**, m715–m716.

[bb10] Oxford Diffraction (2008). *CrysAlis CCD* and *CrysAlis RED* Oxford Diffraction Ltd, Yarnton, England.

[bb11] Petković, M., Petrović, B., Savić, J., Bugarčić, Ž. D., Dimitrić-Marković, J., Momić, T. & Vasić, V. (2010). *Int. J. Mass Spectrom.* **290**, 39—46.

[bb12] Roshal, A. D., Grigorovich, A. V., Doroshenko, A. O., Pivovarenko, V. G. & Demchenko, A. P. (1998). *J. Phys. Chem. A*, **102**, 5907–5914.

[bb13] Roshal, A. D., Munoz, A., Sakhno, T. V. & Boisdon, M.-T. (2002). *Chem. Heterocycl. Compd*, **11**, 1597–1604.

[bb14] Roshal, A. D., Sakhno, T. V., Verezubova, A. A., Ptiagina, L. M., Musatov, V. I., Wróblewska, A. & Błażejowski, J. (2003). *Funct. Mater.* **10**, 419–426.

[bb15] Sheldrick, G. M. (2008). *Acta Cryst.* A**64**, 112–122. 10.1107/S0108767307043930 18156677

[bb16] Spek, A. L. (2009). *Acta Cryst.* D**65**, 148–155. 10.1107/S090744490804362X PMC2631630 19171970

[bb17] Sytnik, A., Gormin, D. & Kasha, M. (1994). *Proc. Natl Acad. Sci. USA*, **91**, 11968–11972. 10.1073/pnas.91.25.11968 PMC45357 7991566

[bb18] Thalladi, V. R., Weiss, H.-C., Bläser, D., Boese, R., Nangia, A. & Desiraju, G. R. (1998). *J. Am. Chem. Soc.* **120**, 8702–8710.

